# Hemispheric asymmetry: Looking for a novel signature of the modulation of spatial attention in multisensory processing

**DOI:** 10.3758/s13423-016-1154-y

**Published:** 2016-09-01

**Authors:** Yi-Chuan Chen, Charles Spence

**Affiliations:** Crossmodal Research Laboratory, Department of Experimental Psychology, University of Oxford, 9 South Parks Road, Oxford, OX1 3UD UK

**Keywords:** Rightward-biased attention, Hemispace, Asymmetry, Crossmodal attention, Prior entry, Multisensory integration

## Abstract

The extent to which attention modulates multisensory processing in a top-down fashion is still a subject of debate among researchers. Typically, cognitive psychologists interested in this question have manipulated the participants’ attention in terms of single/dual tasking or focal/divided attention between sensory modalities. We suggest an alternative approach, one that builds on the extensive older literature highlighting hemispheric asymmetries in the distribution of spatial attention. Specifically, spatial attention in vision, audition, and touch is typically biased preferentially toward the right hemispace, especially under conditions of high perceptual load. We review the evidence demonstrating such an attentional bias toward the right in extinction patients and healthy adults, along with the evidence of such rightward-biased attention in multisensory experimental settings. We then evaluate those studies that have demonstrated either a more pronounced multisensory effect in right than in left hemispace, or else similar effects in the two hemispaces. The results suggest that the influence of rightward-biased attention is more likely to be observed when the crossmodal signals interact at later stages of information processing and under conditions of higher perceptual load—that is, conditions under which attention is perhaps a compulsory enhancer of information processing. We therefore suggest that the spatial asymmetry in attention may provide a useful signature of top-down attentional modulation in multisensory processing.

Our senses are often bombarded by massive amounts of incoming information. Attention, serving as a mechanism of selection, can be oriented endogenously (or voluntarily) to help prioritize those sensory inputs that are critical for our current goals; alternatively, attention can be oriented exogenously (or involuntarily) toward salient sensory signals (e.g., Driver & Spence, 1998; Spence, 2010a; see also the Attention section in Stein, 2012, for a review). The role of attention in modulating multisensory perception has intrigued researchers for more than four decades now. Early research was taken to suggest that attention was biased toward the visual modality, leading to visual dominance over the other senses in human multisensory perception (e.g., Posner, Nissen, & Klein, 1976; see also Spence, Parise, & Chen, 2011, for a more recent review). Subsequently, researchers have addressed the question of whether different sensory modalities share a common attentional control mechanism, as demonstrated by studies of crossmodal spatial orienting (e.g., Spence, 2010a, b, 2014; Spence & Driver, 2004) and by the limits on crossmodal attentional resources that have been identified at a more central stage of information processing (e.g., Arnell & Jolicœur, 1999; Duncan, Martens, & Ward, 1997; Soto-Faraco & Spence, 2002; Soto-Faraco et al., 2002; Wickens, 2002). Finally, researchers have demonstrated that people can selectively attend to the stimuli presented in one sensory modality at the expense of those presented in another (Spence & Driver, 1997b; Spence, Nicholls, & Driver, 2001). Nevertheless, attention may automatically spread to the stimulus presented in another, unattended modality when these crossmodal stimuli are associated in terms of their spatial or temporal coincidence (known as *crossmodal object-based attention*; see Busse, Roberts, Crist, Weissman, & Woldorff, 2005; Turatto, Mazza, & Umiltà, 2005).

One intriguing, but as yet puzzling, question concerns the role of attention in the processing of information from the different senses. One of the problems here is that the effects of attention and multisensory processing^1^ on human perception and performance can be hard to disentangle, because they often lead to similar outcomes (e.g., Shimojo, Watanabe, & Scheier, 2001; Spence & Ngo, 2012). What is more, bidirectional interactions are thought to occur between these two processes. That is, multisensory stimuli may be integrated preattentively, thus giving rise to a salient multisensory event that exogenously captures attention; on the other hand, attention can be endogenously devoted to crossmodal stimuli that might be associated as a single object/event, leading to a more pronounced multisensory integration effect (see De Meo, Murray, Clarke, & Matusz, 2015; Talsma, Senkowski, Soto-Faraco, & Woldorff, 2010, for reviews). In Macaluso et al.’s (2016) recent review, ten factors are summarized, including the characteristics of the stimuli, the task demands, and the capacity of cognitive resources that may determine how attention and multisensory processing interact (see Fig. 2 of Macaluso et al., 2016). For example, strong (i.e., suprathreshold) and salient crossmodal stimuli presented in a simple detection task are more likely to be integrated preattentively, which may, in turn, lead to the exogenous orienting of attention. On the other hand, weak (i.e., near-threshold), complicated, and meaningful stimuli presented in a discrimination or identification task involving decisional processes are more demanding, and therefore it is necessary for the observer to endogenously attend to the stimuli. Macaluso et al.’s summary highlights that the interaction between attention and multisensory processing is complicated and that the two mechanisms are undoubtedly tightly interwoven. To date, our understanding of the relationship between attention and multisensory information processing is based on a heterogeneous collection of attentional manipulations and empirical phenomena.

In the present article, we propose a novel behavioral signature highlighting the modulatory role of top-down attention on multisensory processing. We appeal to the fact that spatial attention is naturally distributed asymmetrically over the two hemispaces within and across the modalities of vision, audition, and touch; specifically, the right hemispace is preferred. In turn, spatial attention can be oriented faster toward the right than toward the left side. This fact ought, presumably, to lead to an asymmetrical effect on multisensory processing when attention is involved, but not on those phenomena of multisensory processing that can be accomplished preattentively. Indeed, Spence and colleagues have previously reported that attentional effects in crossmodal settings tend to be unevenly distributed across the two hemispaces (Spence, Pavani, & Driver, 2000; Spence, Shore, & Klein, 2001), but more recently such asymmetries have seemingly been overlooked by researchers.

Before we review the evidence for spatial attention asymmetrically modulating multisensory perception, it is important to consider whether certain phenomena of multisensory processing might themselves show some kind of spatial bias. Indeed, such asymmetries have been reported previously, though they have mainly been attributed to a particular lateralized cognitive function, such as face or linguistic processing. Specifically, a more pronounced effect of multisensory processing tends to be observed in the *hemispace* contralateral (rather than ipsilateral) to the *hemisphere* that is specialized for a given cognitive function (see Table 1). One example of such an asymmetry comes from studies of the McGurk effect. This classic example of multisensory integration (e.g., Partan & Marler, 1999) occurs when certain pairs of incongruent visual lip movements and auditory speech stimuli are integrated, thus leading to a new percept (McGurk & MacDonald, 1976). The research shows that the McGurk effect occurs more frequently when the visual stimulus (i.e., the lip movements) is presented in the left rather than the right hemispace (Baynes, Funnell, & Fowler, 1994; Diesch, 1995). This asymmetry has been explained in terms of a right-hemisphere advantage for face processing (e.g., Borod et al., 1998; Ellis, 1983; Sergent, Ohta, & MacDonald, 1992).

**Table 1:** Summary of multisensory processing effects demonstrating a spatial asymmetry

Dominant Hemispace	Dominant Hemisphere and the Lateralized Cognitive Function		Multisensory Processing Effect	Studies
Left	Right	Face processing	McGurk effect	Byanes et al. (1994) Diesch (1995)
		Spatial processing	Auditory facilitation of visual localization performance	Takeshima & Gyoba (2014)
Right	Left	Linguistic processing	Auditory facilitation of visual letter identification performance	Takeshima & Gyoba (2014)

Takeshima and Gyoba (2014) recently demonstrated a larger auditory facilitation resulting from the presentation of a simultaneous tone on visual localization performance in the left as compared to the right hemispace. Their suggestion was that this asymmetry could be attributed to the right hemisphere being specialized for the processing of spatial information (Kimura, 1969; Umiltà et al., 1974). On the other hand, the auditory facilitation elicited by a simultaneously - presented tone on visual letter identification performance was shown to be more pronounced when the letter was presented in the right (rather than the left) hemispace. Their suggestion was that this result reflects the left hemisphere’s specialization for linguistic processing (e.g., Geffen, Bradshaw, & Nettleton, 1972; Kimura, 1961; MacKain, Studdert-Kennedy, Spieker, & Stern, 1983; Scott, Blank, Rosen, & Wise, 2000).^2^ Therefore, when proposing that any asymmetrical effect of multisensory processing can be attributed to spatial attention, such alternative explanations of the results will obviously need to be ruled out first, especially those leading to an expected advantage in the right hemispace.

## Outline of the article

The revived behavioral signature that we propose when trying to assess the modulatory role of attention on multisensory processing is linked to the fact that spatial attention, in the majority of cases, is preferentially biased toward the *right* side in humans (e.g., Hämäläinen & Takio, 2010). Any such rightward bias of attention should modulate any multisensory processing requiring attention, in terms of an effect that is prioritized (i.e., occurs earlier in time) or more pronounced in magnitude when the stimuli happen to be presented in the right rather than the left hemispace.

We start by reviewing previous studies that have addressed the question of whether and how attention modulates multisensory processing. We then go on to review the human behavioral evidence that has suggested a rightward bias in unimodal visual, auditory, and tactile attention. We review the three possible mechanisms that have been put forward over the years to account for this phenomenon. In the following section, we highlight the evidence suggesting that the rightward attentional bias also extends to crossmodal settings, as demonstrated by the results of Spence, Shore, and Klein’s (2001) study of multisensory prior entry, and by Spence et al.’s (2000) crossmodal endogenous-orienting study. Next, we reanalyze the data reported previously by Chen and Spence (2011), demonstrating larger crossmodal facilitation on visual letter identification performance when the visual stimuli are presented in the right rather than the left hemispace. Additionally, published research that has failed to show any such asymmetry in multisensory processing between the two hemispaces is also reviewed, and the possible implications of these null results are discussed.

On the basis of the literature that has been published to date, and that is reviewed here, we suggest that utilizing the fundamental rightward bias in spatial attention in the future can extend our understanding of those conditions under which attention is involved in multisensory processing. In turn, investigating this issue contributes to an evaluation of whether attention is a domain-general mechanism that similarly modulates the processing of the sensory signals coming from either a single or multiple modalities (e.g., Klemen & Chambers, 2012; van Atteveldt, Murray, Thut, & Schroeder, 2014). Finally, such knowledge of multisensory attention can potentially be applied in the field of ergonomics, such as by developing multisensory warning systems to improve people’s information processing and decision making under highly demanding conditions (e.g., Baldwin et al., 2012; Ho & Spence, 2008; Ngo, Pierce, & Spence, 2012).

## Review of the attentional modulation of multisensory processing

Researchers have utilized various attentional manipulations to examine whether or not a particular instance, or type, of multisensory processing is modulated by attention. For example, participants can fully attend to a primary task that involves multisensory processing, or else they can choose to divide their attention between the primary task and another, secondary task involving the unimodal stimuli. Researchers then compare people’s multisensory performance under single- versus dual-task conditions. The McGurk effect, for instance, occurs more frequently under conditions of single than of dual tasking (see Alsius, Möttönen, Sams, Soto-Faraco, & Tiippana, 2014; Alsius, Navarra, Campbell, & Soto-Faraco, 2005; Alsius, Navarra, & Soto-Faraco, 2007). Such results have been taken to suggest that audiovisual speech perception is modulated by attention (see also Fairhall & Macaluso, 2009; Fernández, Visser, Ventura-Campos, Ávila, & Soto-Faraco, 2015, for evidence from neuroimaging studies).

When the signals from different sensory modalities provide redundant cues along a certain dimension (known as *amodal* features, such as space and size in vision and touch), it has been suggested that these cues are integrated in a manner that is statistically optimal in human behavior (i.e., following Bayes’s rule). Specifically, the weighting of a component signal in the outcome of multisensory integration is positively correlated with its reliability (Alais & Burr, 2004; Ernst & Banks, 2002; Gori, Sandini, & Burr, 2012; Körding et al., 2007). Later studies have also observed that Bayes’s rule can be used to explain the activities at the single- and the group-neuronal levels in multisensory processing (e.g., Fetsch, Pouget, DeAngelis, & Angelaki, 2012; Rohe & Noppeney, 2015, 2016). Nevertheless, to date, the role of attention in such optimal multisensory integration remains unclear. For example, by adding a secondary visual task that is irrelevant to the primary task of visuotactile processing (i.e., dual tasking), optimal integration was maintained and the weighting of the visual input was similar under both single- and dual-task conditions (Helbig & Ernst, 2008; Wahn & König, 2016). By contrast, when the secondary task involved the auditory stimulus that might be integrated with a visual stimulus in the primary task, the reliability of that auditory signal, and so its weighting, was enhanced by attention (Vercillo & Gori, 2015). However, note that the latter study failed to verify whether the participants’ performance was better explained by optimal integration or sensory dominance.

Another type of attentional manipulation has utilized the fact that participants’ attention can be selectively focused on a specific sensory modality or can be distributed over multiple modalities (e.g., Spence & Driver, 1997b; Spence, Nicholls, & Driver, 2001). Indeed, several studies have demonstrated a more pronounced multisensory effect when both of the to-be-integrated stimuli were attended than when only one of them was attended. For example, in the task of discriminating red versus blue presented in the visual modality (color patches) and/or in the auditory modality (spoken words), people’s response times (RTs) were faster than the statistically estimated RT based on the race model (Miller, 1982) only when the participants attended to both modalities, rather than selectively to either one of them (Mozolic, Hugenschmidt, Peiffer, & Laurienti, 2008). In an event-related potential study, the neural activities elicited by audiovisual stimuli were larger than the sum of those elicited by unimodal visual and auditory stimuli, and the earliest difference was observed at 55 ms after stimulus onset (called the P50 component); however, such a P50 effect was observed only when both modalities were attended (Talsma, Doty, & Woldorff, 2007). Most recently, Odegaard, Wozny, and Shams (2016) examined the influence of attention on audiovisual integration in a spatial task (the spatial-ventriloquism effect; Jackson, 1953) and a temporal task (the sound-induced flash illusion; Shams, Kamitani, & Shimojo, 2000, 2002). After the participants’ performance was fitted with Bayesian models (Körding et al., 2007; Wozny, Beierholm, & Shams, 2008), the results revealed that the reliability of the visual and/or auditory signal was higher in the condition in which attention was focused on that modality than when dividing attention between both modalities. However, there was no significant change in the tendency to bind the visual and auditory signals.

### Does spatial attention modulate multisensory processing?

Researchers have demonstrated extensive crossmodal links in spatial attention between the modalities of vision, audition, and touch (e.g., McDonald, Teder-Sälejärvi, & Hillyard, 2000; Spence & Driver, 1996, 1997a; Spence et al., 2000; see Spence, 2014, for a review). Nevertheless, it would seem fair to say that the question of whether spatial attention modulates multisensory processing has not, as yet, reached a consensual answer among researchers (see Santangelo & Macaluso, 2012, for a review). For example, in one series of spatial-ventriloquism studies showing that a sound may be mislocalized toward the location of a spatially discrepant visual stimulus (Alais & Burr, 2004; Bertelson & Radeau, 1981; Jackson, 1953), the participants’ spatial attention was oriented either toward or away from the visual stimulus. The results of several such studies have demonstrated that the audiovisual spatial-ventriloquism effect is *not* susceptible to the manipulation of a participant’s spatial attention, when it is oriented either endogenously or exogenously (Bertelson, Vroomen, de Gelder, & Driver, 2000; Vroomen, Bertelson, & de Gelder, 2001). By contrast, it has been suggested that spatial ventriloquism can be modulated by the manipulation of a participant’s visual perceptual load, at least when measured by its aftereffects.^3^ A larger ventriloquism aftereffect was observed when the participants’ perceptual load was higher during the adaptation phase (see Eramudugolla, Kamke, Soto-Faraco, & Mattingley, 2011).

In another series of studies, Santangelo and his colleagues demonstrated that a peripheral crossmodal cue (either audiovisual or audiotactile) exogenously captured their participants’ attention even when it was putatively focused on a highly demanding central visual task (Ho, Santangelo, & Spence, 2009; Santangelo, Ho, & Spence, 2008; Santangelo & Spence, 2007; see Spence, 2010b, for a review). By contrast, the component unimodal cues (visual, auditory, or tactile), when presented individually, were rendered entirely ineffective in terms of capturing participants’ spatial attention under such highly demanding conditions. The suggestion that has emerged from this line of research is that simultaneously - presented multisensory inputs (even when they are not precisely co-located in space; see Spence, 2010b, 2013, for reviews) are integrated in an automatic and preattentive manner. In turn, such multisensory events capture an observer’s attention due to their saliency (e.g., van der Burg, Olivers, Bronkhorst, & Theeuwes, 2008; van der Burg, Talsma, Olivers, Hickey, & Theeuwes, 2011; see Santangelo & Spence, 2008; Talsma et al., 2010, for reviews). By contrast, van der Burg, Olivers, and Theeuwes (2012) later demonstrated that such exogenous attentional capture by multisensory events can be modulated by the spatial distribution of a participant’s attention (either focused in the center or distributed over a broad area). In summary, then, the empirical results that have been published to date appear to provide only very weak evidence for spatial attention having a modulatory effect on multisensory processing, though other types of attentional manipulation (such as perceptual load or attentional distribution) might modulate multisensory processing in the same task.

## Hemispheric asymmetry: Rightward biasing of visual, auditory, and tactile attention

The asymmetry of spatial attention has long been demonstrated in the neurological disorder known as *extinction*, which often occurs in those patients suffering from contralateral spatial neglect following right parietal and/or frontal-lobe damage (Berlucchi, Aglioti, & Tassinari, 1997; Weintraub & Mesulam, 1987; see Behrmann & Shomstein, 2015; Humphreys & Bruce, 1989; Mesulam, 1999; Singh-Curry & Husain, 2010, for reviews). Patients with extinction are often unable to detect (i.e., they lack an awareness of) those visual stimuli that happen to be presented in the left hemispace when other stimuli are simultaneously - presented in the right hemispace. They are, however, able to respond accurately to visual stimuli presented unilaterally, in either the left or the right hemispace. Hence, the phenomenon of extinction cannot be attributed to any deficit in early visual sensory processing (e.g., Brain, 1941; Paterson & Zangwill, 1944). Extinction is often reported in the left hemispace, whereas the similar symptoms in the right hemispace following left parietal and/or frontal-lobe damage are typically milder and more likely to dissipate (e.g., Stone et al., 1991).

The phenomenon of extinction has not only been demonstrated in the visual modality; it also occurs in both audition (e.g., De Renzi, Gentilini, & Pattacini, 1984; Heilman & Valenstein, 1972; see Clarke & Thiran, 2004, for a review) and touch (e.g., Beschin, Cazzani, Cubelli, Della Sala, & Spinazzola, 1996; Moscovitch & Behrmann, 1994; Pierson-Savage, Bradshaw, Bradshaw, & Nettleton, 1988; Schwartz, Marchok, Kreinick, & Flynn, 1979). The existence of extinction in the three spatial modalities (i.e., vision, audition, and touch) hints that perhaps a common spatial attentional mechanism is biased toward the right side of space (though see Sinnett, Juncadella, Rafal, Azañón, & Soto-Faraco, 2007).

### Rightward-biased attention in neurologically normal participants

Relevant to the argument that we wish to make here, an attentional advantage in the right hemispace has also been reported in healthy adults (e.g., Heilman & van den Abell, 1979; see Hämäläinen & Takio, 2010, for a review). Supporting evidence has come, for example, from a study by Railo, Tallus, and Hämäläinen (2011) showing that a gray disc is rated as having higher visibility when it is presented on the right rather than the left. In this case, the researchers suggested that the effect resulted from rightward-biased attention facilitating the perceived luminance contrast of the visual stimuli (see Carrasco, Ling, & Read, 2004). In addition, a mild extinction-like effect in neurologically - normal adults has also been reported: When two visual targets are presented, one in either hemispace, participants are more likely to detect or localize the target presented on the right than the one presented on the left. Furthermore, such extinction-like phenomena have been observed more frequently in children and the elderly, whose attentional capacity and control are limited relative to what is seen in healthy adults (Takio, Koivisto, Tuominen, Laukka, & Hämäläinen, 2013; though see Goodbourn & Holcombe, 2015).

In the auditory modality, the rightward biasing of spatial attention has been demonstrated in dichotic-listening studies: When two strings of syllables are presented to each ear, participants often preferentially report those syllables that have been presented to the right, rather than to the left, ear (Kimura, 1961, 1964, 1967; see Hugdahl et al., 2009, for a review). That the right-side advantage in audition occurs at a perceptual, rather than a sensory, level has been shown by presenting the sounds from free-field loudspeakers instead of over headphones (Bertelson, 1982; Morais, 1978; Pierson, Bradshaw, & Nettleton, 1983). Given that speech stimuli were used in the above studies, the conventional view that linguistic processing is lateralized in the left hemisphere provides an alternative explanation for such an advantage in the right hemispace (Geffen et al., 1972; Kimura, 1961; MacKain et al., 1983). To rule out this alternative possibility, the pure tones used in a simple detection task still demonstrated a higher accuracy when the tone was presented on the right rather than on the left side (Takio, Koivisto, Laukka, & Hämäläinen, 2011). Similar to the rightward bias that has been shown for visual attention (Takio et al., 2013), this rightward bias in the distribution of auditory attention is apparently also more pronounced in children and the elderly than in adults (Takio et al., 2009, 2011).

Finally, a rightward attentional bias has also been demonstrated in the tactile modality. For example, participants’ RTs to detect vibrotactile stimuli presented to either the left or the right hand are faster when that hand is placed in the right rather than the left hemispace relative to the body midline (Bradshaw, Bradshaw, Pierson-Savage, & Nettleton, 1988; Bradshaw, Nathan, Nettleton, Pierson, & Wilson, 1983; Bradshaw & Pierson, 1985). Note that in these experiments, the participants’ heads and gaze were also manipulated toward or away from the possible location of the tactile stimulus, which led to the necessity of coordination between the visual and tactile spatial frames of reference. The results demonstrated that the external spatial frame associated with the visual modality was dominant and was utilized for visuotactile spatial coordination (Bradshaw et al., 1988).

#### High perceptual load leads to rightward-biased attention

Takio et al. (2009, 2011, 2013) have reported that the effect of rightward-biased attention in the visual and auditory modalities is more pronounced in children and the elderly than in healthy adults. This difference may well be attributable to the fact that attentional capacity is smaller in the former groups. In healthy adults, rightward-biased attention can be induced by increasing the perceptual load of the task. For example, when researchers add a secondary unimodal visual or auditory task, participants’ performance in the primary visual task demonstrates an advantage for target(s) presented in the right as compared to the left hemispace (Peers, Cusack, & Duncan, 2006; Pérez et al., 2009). Such a result was similar to the performance of patients with damage to the right parietal lobe in the same study, though the effect was milder in the healthy adults (Peers et al., 2006).

Eramudugolla et al. (2011) demonstrated an effect of rightward-biased attention in the study of the crossmodal ventriloquism aftereffect: During the adaptation phase, in addition to the spatially discrepant visual and auditory stimulus pair used for inducing the ventriloquism effect, a series of central visual patterns were also presented as part of an additional visual detection task. The participants’ perceptual load was manipulated by designating that the target was either a simple pattern (low-load condition) or multiple complex patterns (high-load condition). The results demonstrated that a significantly larger ventriloquism aftereffect was observed in the high- than in the low-load condition when the sound was realigned toward the right side; nevertheless, such a difference was only observed in right rather than in left hemispace. Eramudugolla et al. therefore demonstrated that by increasing the perceptual load, an attentional modulation of multisensory processing can be revealed asymmetrically (in the right, but not in the left, hemispace).

### Mechanisms underlying the rightward attentional bias

Over the years, at least three possible mechanisms have been put forward to account for the rightward bias in spatial attention. The first two are neural models based on lateralization and interactions between the two hemispheres. These two models still need further evidence to verify whether one of them is more comprehensive, or whether instead they operate hierarchically (e.g., Duecker & Sack, 2015; Scolari, Seidl-Rathkopf, & Kastner, 2015). The third model provides an ecological perspective, according to which the biasing of people’s spatial attention toward the right results from an adaptation to their interaction with the outside world. These three models should nevertheless not necessarily be considered mutually exclusive.

#### The right-hemisphere specialization model

Dominant among these models is the *right-hemisphere specialization model* of spatial attention (see Fig. 3 of Mesulam, 1999). This model is based both on evidence from neglect patients and on psychophysiological studies of visual perception in healthy participants. Specifically, the left hemisphere mainly coordinates the distribution of attention in the right hemispace and directs attention rightward; on the other hand, the right hemisphere coordinates the distribution of attention in both hemispaces and directs attention toward either side in a more evenly balanced manner (see also Corbetta & Shulman, 2002; Heilman & van den Abell, 1980; Iturria-Medina et al., 2011). According to this model, both hemispheres control attention in the right hemispace, so that one may substitute for the other if either happens to be engaged temporally, or to be damaged following stroke (see also Hämäläinen & Takio, 2010). Subsequently, a rightward biasing of spatial attention has been demonstrated under conditions of high (as compared to low) perceptual load in both healthy adults and patients with parietal lesions (Eramudugolla et al., 2011; Peers et al., 2006; Pérez et al., 2009).

#### The interhemispheric competition model

According to the second model, the *interhemispheric competition model*, each hemisphere only directs attention to the contralateral hemispace, and hence the distribution of spatial attention toward either side is thought to be controlled by reciprocal inhibition between the two hemispheres (e.g., Cohen, Romero, Servan-Schreiber, & Farah, 1994; Kinsbourne, 1970; Posner, Walker, Friedrich, & Rafal, 1987). A slight imbalance in inhibition—in most cases, stronger inhibition from the left than from the right hemisphere—is then thought to result in the rightward attentional bias (Szczepanski & Kastner, 2013).

#### The handedness preference model

According to this last model, the *handedness preference model*, the bias results from a developmental consequence of a right-side preference for motor planning and control. A preference for head turning toward the right and using the right hand (such as thumb sucking) has been demonstrated in infancy, even in utero (Ginsburg, Fling, Hope, Musgrove, & Andrews, 1979; Hepper, Shahidullah, & White, 1991; Turkewitz, Gordon, & Birch, 1965). Some researchers have further suggested that later on, this bias might lead to the emergence of right handedness (Coryell, 1985; Michel, 1981).

In adults, a rightward attentional bias has been reliably observed in right-handers (Le Bigot & Grosjean, 2012; Lloyd, Azañón, & Poliakoff, 2010; Railo et al., 2011; though see Szczepanski & Kastner, 2013), since this is the dominant side on which right-handers are used to interacting with the outside world. This hypothesis seems plausible; however, left-handers fail to demonstrate a reliable leftward bias in attention, even when the left- and right-handers who were tested had matched handedness scores (Le Bigot & Grosjean, 2012; Railo et al., 2011; though see Kerr, Mingay, & Elithorn, 1963). One possible explanation is that left-handers often use their left and right hands similarly well in a single-handed task, and therefore they can flexibly distribute spatial attention to either hemispace. In sum, the rightward bias of spatial attention is correlated with the fact that most people are right-handed.

## The rightward bias in crossmodal spatial attention

The results reviewed above clearly highlight a rightward attentional bias in the visual, auditory, and tactile modalities when they are studied individually. Given the existence of extensive crossmodal links in spatial attention between these three senses (see Spence, 2010a, 2014, for reviews), it would seem only natural to assume that rightward-biased attentional effects should also be observed in the case of multisensory processing, as well. Indeed, neuropsychological evidence from patients has demonstrated that extinction occurs between stimuli that happen to be presented in different sensory modalities. For example, a tactile stimulus presented to a patient’s left hand may well go undetected in some proportion of trials when a visual stimulus happens to be presented somewhere close to the patient’s right hand at around the same time (di Pellegrino, Làdavas, & Farnè, 1997; Mattingley, Driver, Beschin, & Robertson, 1997; Rapp & Hendel, 2003).^4^

**Fig. 1:**
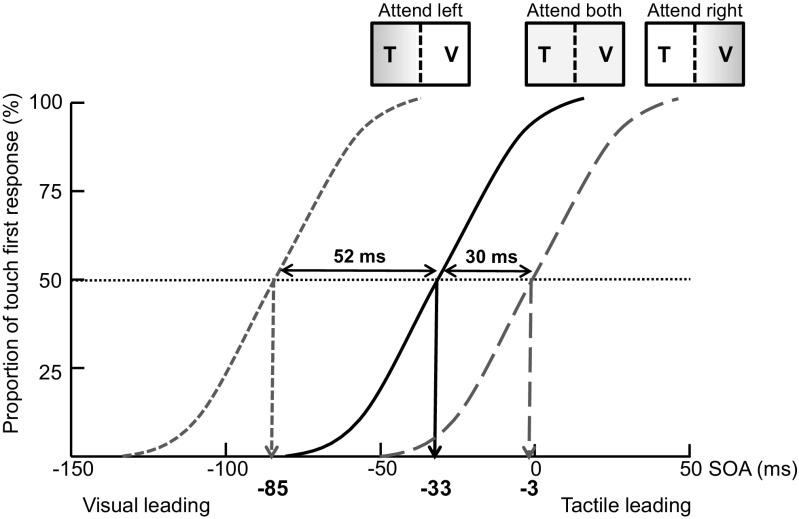
Schematic representation of the multisensory prior-entry effect reported by Spence et al. (2001). The proportion of “touch first” responses increased as a function of stimulus onset asynchrony (SOA; negative values indicate the visual-leading conditions, and positive values, the tactile-leading conditions), giving rise to a cumulative curve. In this curve, the 50 % “touch first” responses point corresponds to the SOA at which participants presumably perceived the two stimuli as simultaneous (i.e., the point of subjective simultaneity [PSS]). When one of the stimuli happened to be attended, it would be perceived as having been presented earlier in time than the other stimulus, thus leading to a shift of the PSS. Here we demonstrate an example in which the visual stimulus was presented on the right side and the tactile stimulus on the left (one of the conditions in their Exp. 4). The gray shading represents how the participants’ spatial attention was distributed. As compared to the baseline condition in which the participants divided their attention between both sides (black solid line), the shift of the PSS was smaller when the participants attended to the *right* (gray dashed line; the visual stimulus was attended, and therefore the PSS shifted toward the tactile-leading condition) than when the participants attended to the *left* (gray dotted line; the tactile stimulus was attended, so the PSS shifted toward the visual-leading condition). V, visual stimulus; T, tactile stimulus

Spence, Shore, and Klein’s (2001) study of multisensory prior entry provided evidence of rightward-biased attention modulating multisensory perceptual performance in healthy adults. *Prior entry* refers to the phenomena that when a stimulus is attended, it is perceived to have been presented earlier in time than another stimulus that is presented simultaneously but is unattended (Titchener, 1908; Zampini, Shore, & Spence, 2005; see Spence & Parise, 2010, for a review). For example, in a series of temporal-order judgment experiments reported by Spence et al. (2001), pairs of visual and tactile stimuli were presented to left and/or to right hemispace (27° into the periphery) at various stimulus onset asynchronies (SOAs). The participants had to report the modality of the first stimulus. The point of subjective simultaneity (PSS) corresponded to the SOA at which participants reported 50 % “touch first” responses (i.e., the participants were assumed to perceive crossmodal simultaneity). The prior-entry effect is indexed by the magnitude of the shift in the PSS: Given that attention speeds up the relative time of arrival of an attended, as compared to an unattended, stimulus (Vibell, Klinge, Zampini, Spence, & Nobre, 2007), the unattended stimulus therefore needs to be presented even earlier in time for perceptual simultaneity to be achieved (see Fig. 1 for an example of a condition in which a tactile stimulus is presented on the left side and a visual stimulus on the right side).

First, Spence, Shore, and Klein (2001) measured baseline performance under those conditions in which the visual and tactile stimuli were presented on either side with equal probability, and presumably the participants’ attention was divided spatially (Exp. 1). In subsequent experiments, the participants’ attention was endogenously oriented toward either the right or the left side on a block-by-block basis. That is, the majority of the visual and tactile stimuli in a particular block of trials were presented either on the left or on the right side. In half of the trials, the visual and tactile stimuli were presented on opposite sides, and this was the condition in which multisensory prior entry was documented (Exps. 3 and 4). Note that the participants’ responses (reporting either “visual first” or “tactile first”) were independent of the side (left or right) to which attention had been oriented endogenously. Interestingly, the multisensory prior-entry effect was smaller when the participants endogenously attended to the right than to the left when the divided-attention condition served as the baseline. The smaller prior-entry effect in the attend-right than in the attend-left condition was observed when calculating the amount of PSS shift across Experiments 1 and 3 (when combining the conditions of both tactile/left–visual/right and tactile/right–visual/left, attend right = 3 ms, attend left = 38 ms) or within Experiment 4 on a within-participant basis (attend right = 29 ms, attend left = 38 ms). According to Spence et al. (2001), the smaller difference between the divided-attention and attend-right conditions than between the divided-attention and attend-left conditions could be attributed to the fact that participants’ attention was naturally biased toward the right side of space in the divided-attention condition. The results of this study therefore provide an example that people’s rightward-biased attention can selectively enhance the perception of the stimulus presented on the right in a crossmodal setting.

Spence et al. (2001) also reported another result consistent with a natural bias of attention between sensory modalities: Posner et al. (1976) proposed that people’s attention is preferentially directed toward vision rather than other modalities, which leads to visual-dominance phenomena (e.g., Colavita, 1974). Consistently, Spence et al. (2001) observed a smaller prior-entry effect when the participants were induced to attend to vision rather than to touch. Specifically, when the condition in which the participants divided their attention to both vision and touch served as the baseline, the PSS shift in the attend-vision condition (31 ms) was smaller than that in the attend-touch condition (102 ms). Taken together, in their multisensory prior-entry study, Spence et al. (2001) demonstrated two effects caused by the natural biasing of attention. That is, people’s attention tended to be biased toward vision rather than touch and, critically, toward the right rather than the left side of space.

However, an alternative explanation needs to be excluded here: The smaller prior-entry effect on the right than on the left side might be due to it being harder for the participants to orient their attention endogenously toward the right than toward the left. The rightward-biased attention observed by Spence et al. (2000) also helps rule out this possibility. In their Experiment 3, an orthogonal-cuing design was used: The participants’ attention was endogenously oriented toward either the left or the right by a central arrow cue, and they had to discriminate the presentation of a visual or a tactile target at a higher or a lower elevation. The results demonstrated an overall shorter RT for right than for left targets (494 vs. 512 ms; see also Bradshaw & Pierson, 1985), suggesting an advantage for orienting attention toward right rather than left hemispace. Taken together, the fact that spatial attention is naturally biased toward right hemispace likely leads to the similar performance in the baseline and attend-right conditions (as compared to the attend-left condition), as well as faster orienting responses toward right than toward left hemispace.

## Does rightward-biased attention lead to an asymmetrical effect on multisensory processing?

In the previous section, the results of studies of crossmodal extinction in patients and of two crossmodal attention studies reported by Spence and colleagues (Spence et al., 2000; Spence et al., 2001) were reviewed. These results suggest that attention is biased toward the right hemispace in multisensory settings. In this section, we evaluate whether an advantage in the right over the left hemispace can be observed if attention serves as a top-down modulation in multisensory processing. To do so, we reanalyze the data from Chen and Spence’s (2011, Exps. 1 and 5) study of the crossmodal facilitation of visual identification performance.

### Prioritized crossmodal facilitation in right as compared to left hemispace in the backward-masking paradigm

Chen and Spence (2011) adopted a backward-masking paradigm in which two letters (i.e., the target and mask) were presented sequentially but overlapped spatially.^5^ In Experiment 1, three factors were manipulated: Sound (present or absent), Interstimulus Interval (ISI: 0, 13, 27, 40, 80, and 133 ms), and Hemispace (left or right). The pure tone was randomly presented on half of the trials. Participants were informed that on some trials they might hear a beep, and that if the sound was present, it would always accompany the first letter (i.e., the target). A pure tone was presented simultaneously, and with equal amplitudes, from four loudspeaker cones placed at the four corners of the monitor (Left/Right × Top/Bottom). Given the small spatial disparity between the visual and auditory stimuli (within 10°), the perceived location of the sound should have been ventriloquized toward the location of visual stimulus (Jackson, 1953).

Six ISIs between the target and mask were presented to demonstrate the masking effect, which typically shows up as the participants’ accuracy in target identification monotonically increasing with increasing ISI. This is because the presentation of the mask letter would be less likely to interfere with processing of the target letter as the blank interval between them increased. The target and mask were always presented from the same spatial location, randomly on either the left or the right (7.17° from central fixation), to avoid participants fixating the target location.

**Fig. 2:**
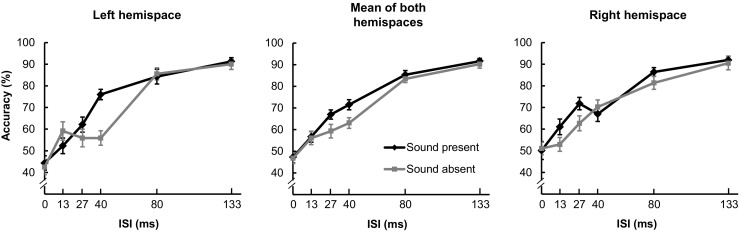
Mean accuracies of participants’ letter identification performance in Chen and Spence’s (2011) Experiment 1. The central panel represents the results combining the two hemispaces that were reported by Chen and Spence. The right and left panels represent the results of a reanalysis of the data, separating those conditions in which the visual target was presented in right or left hemispace, respectively. The error bars represent ±1 standard error of the means

In Chen and Spence’s (2011) Experiment 1 (see the central panel in Fig. 2), 17 participants remained in the final analysis. The accuracy data were subjected to arcsine transformation in order to linearize the percentage data. The data were submitted to a two-way analysis of variance (ANOVA) with the factors Sound and ISI, while the third factor, Hemispace, was collapsed. Crossmodal facilitation by sound was only observed at the ISIs of 27 and 40 ms.

In a new data analysis, the transformed data were submitted to a three-way ANOVA with the factors Sound, ISI, and Hemispace. Critically, the three-way interaction was significant [*F*(5, 80) = 6.25, *MSE* = 0.05, *p* < .001, *η*_p_^2^ = .28]. Two two-way follow-up ANOVAs with the factors Sound and ISI were then conducted for the right and left hemispaces, separately. In the right hemispace (the right panel in Fig. 2), the interaction between sound and ISI was significant [*F*(5, 80) = 2.28, *MSE* = 0.05, *p* = .05, *η*_p_^2^ = .13]. Post-hoc *t* tests demonstrated that only at the 27-ms ISI was the accuracy higher in the sound-present than in the sound-absent condition (*p* < .008). In the left hemispace (the left panel in Fig. 2), the interaction between sound and ISI was significant as well [*F*(5, 80) = 6.15, *MSE* = 0.06, *p* < .001, *η*_p_^2^ = .28]. Post-hoc *t* tests demonstrated that the accuracy was higher in the sound-present than in the sound-absent condition only at the 40-ms ISI (*p* < .001). These results suggest that crossmodal facilitation occurred at a shorter ISI (i.e., it was prioritized) when the target letter was presented in the right rather than the left hemispace. Note that the main effect of hemispace was not significant [*F*(1, 16) = 0.86, *MSE* = 0.48, *p* = .37, *η*_p_^2^ = .05], suggesting that letter identification was not significantly better in the right than in the left hemispace. This result therefore indicates no advantage for letter identification in the right over the left hemispace in our design (cf. Takeshima & Gyoba, 2014).

In Experiment 5, 23 participants remained in the final analysis. Three factors were manipulated: Sound Location (consistent, inconsistent, and sound absent), ISI (0, 13, 27, 40, 80, and 133 ms), and Hemispace (right or left). The pure tone was presented simultaneously from the two speakers on the same side as the visual target (left or right) in one third of the trials (*spatially consistent*), presented from the two speakers on the other side of the visual target in one third of the trials (*spatially inconsistent*), or only the visual target was presented in the rest of the (*sound-absent*) trials. Note that in the spatially inconsistent condition, the perceived location of the sound, if any, might be ventriloquized to the center rather than farther toward the location of the target letter (see Bonath et al., 2007).

**Fig. 3:**
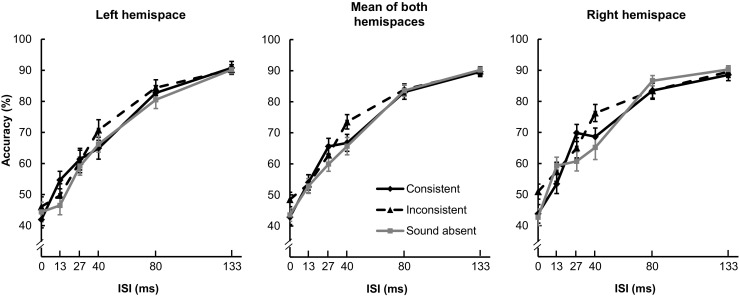
Mean accuracies of participants’ letter identification performance in Chen and Spence’s (2011) Experiment 5. The central panel represents the results combining the two hemispaces that were reported by Chen and Spence. The right and left panels represent the results of a reanalysis of the data separating those conditions in which the visual target was presented in right or left hemispace, respectively. In this experiment, the spatial consistency between the visual target and the sound was manipulated. The error bars represent ±1 standard error of the means

In Chen and Spence’s (2011) Experiment 5 (the central panel in Fig. 3), the accuracy data were transformed and submitted to a two-way ANOVA with the factors Sound Location and ISI, while the Hemispace factor was collapsed. The results demonstrated that the presentation of a simultaneous sound, from either the consistent or the inconsistent location, elicited a crossmodal facilitation effect when compared to the sound-absent condition; nevertheless, the crossmodal facilitation induced by the presentation of the spatially consistent sound (27-ms ISI) occurred at a shorter ISI than that induced by the spatially inconsistent sound (40-ms ISI).

Next, the accuracy data were transformed and submitted to a three-way ANOVA on the factors Sound Location, ISI, and Hemispace. The three-way interaction was significant [*F*(10, 220) = 2.27, *MSE* = 0.05, *p* < .05, *η*_p_^2^ = .09]. Two two-way follow-up ANOVAs on the factors Sound Location and ISI were then conducted for the right and left hemispaces, separately. In the right hemispace (the right panel in Fig. 3), there was a significant interaction between sound location and ISI [*F*(10, 220) = 2.64, *MSE* = 0.06, *p* < .01, *η*_p_^2^ = .11]. A one-way ANOVA conducted on the factor Sound Location was significant at the 27-ms [*F*(2, 44) = 5.71, *MSE* = 0.04, *p* < .01, *η*_p_^2^ = .21] and 40-ms [*F*(2, 44) = 5.27, *MSE* = 0.07, *p* < .01, *η*_p_^2^ = .19] ISIs. Post-hoc *t* tests (with Bonferroni corrections) demonstrated that, at the 27-ms ISI, the accuracy of participants’ responding was higher in the consistent than in the sound-absent condition (*p* < .05); at the 40-ms ISI, accuracy was higher in the inconsistent than in both the consistent and sound-absent conditions (*p*s < .05). In the left hemispace (the left panel in Fig. 3), by contrast, neither the main effect of sound location nor the interaction was significant (*F*s < 2.56, *p*s > .09). In summary, then, the crossmodal facilitation effect reported by Chen and Spence (2011, Exp. 5) derived mainly from the condition in which the visual stimuli were presented in right rather than left hemispace. Again, the main effect of hemispace was not significant [*F*(1, 22) = 1.38, *MSE* = 0.49, *p* = .25, *η*_p_^2^ = .06], indicating that no right-hemispace advantage for letter identification was observed.

In Chen and Spence’s (2011) study, the crossmodal facilitatory effect was accounted for by the notion that the simultaneously - presented tone and visual target were bound together to form a multisensory object representation (see Chen & Spence, 2011, pp. 1797–1799; see also Busse et al., 2005). In turn, such multisensory object representations are likely to be consolidated better than unimodal visual object representations, and thus, are less likely to be interrupted by the subsequently presented mask (see also Murray et al., 2004). Critically, the backward masking occurring at the level of the object representation is modulated by attention (e.g., Di Lollo, Enns, & Rensink, 2000; Enns & Di Lollo, 2000).

After reanalyzing the data from Experiments 1 and 5 in Chen and Spence (2011), by separating the hemispaces in which the visual target was presented (i.e., right or left), the results demonstrated that the crossmodal facilitatory effect was either prioritized or only significant in right rather than the left hemispace. Our contention here is that such an advantage of audiovisual integration in the right over the left hemispace ought to be attributed to the rightward biasing of participants’ spatial attention. This might be either the result of crossmodal spatial attention naturally being preferentially distributed toward the right, as was suggested by Spence et al. (2001), or of attention being naturally rightward biased, leading to faster orienting toward the right than toward the left (Spence et al., 2000; see the model presented in Mesulam, 1999). In the latter case, for example, it has been suggested that when a visual target is paired with a simultaneously presented sound (though not necessarily a sound coming from exactly the same spatial location), the two would form a salient multisensory event that is capable of capturing attention and, in turn, enhancing the processing of the event itself (e.g., Santangelo & Spence, 2007; van der Burg et al., 2011; see Talsma et al., 2010, for a review).^6^ Currently, it is difficult to tease apart these two possible attentional mechanisms. Either way, rightward-biased attention would better facilitate the perception of audiovisual events presented in right as compared to left hemispace if attention is involved in multisensory processing. In sum, the results of the reanalysis of the data from Chen and Spence’s (2011) study suggests that the rightward biasing of spatial attention can prioritize, or enhance, the processing of visual and auditory information in right versus left hemispace.

We suggest that such an advantage of multisensory integration in the right hemispace cannot simply be attributed to the lateralization of linguistic or temporal processing, because both should lead to a general advantage in right over left hemispace. The former possibility, regarding linguistic processing, was raised because of the conventional view that the visual targets were letters, and visual information presented in the right hemispace projects to the left hemisphere, which is specialized for linguistic processing (Geffen et al., 1972; Zangwill, 1960). The latter possibility, regarding temporal processing, was raised following evidence showing that visual temporal resolution is higher in the left hemisphere (projection from the right hemispace) than in the right hemisphere (projection from the left hemispace; see Nicholls, 1996; Okubo & Nicholls, 2008).

Such lateralized cognitive functions can easily explain the crossmodal facilitatory effects reported by Takeshima and Gyoba (2014) using the attentional blink (AB) paradigm: The facilitation in the letter identification task was only observed in the right hemispace, whereas the facilitation in the spatial localization task was only observed in the left hemispace. Critically, both facilitatory effects were observed over a wide temporal window, rather than being specific to the time window in which the AB was most pronounced. Takeshima and Gyoba therefore explained their results in terms of a general facilitation of linguistic processing in right hemispace (left hemisphere), and of spatial processing in left hemispace (right hemisphere). By contrast, our results demonstrated that crossmodal facilitation was observed only in a particular time window (i.e., at the 27- and 40-ms ISIs) during which the visual target and mask just happened to be temporally segregated as two events (see Exp. 1 in Chen & Spence, 2011). This result is consistent with the suggestion that multisensory integration occurs after unimodal perceptual grouping/segregation has been completed, so that the crossmodal signals can be clearly mapped and integrated (Spence & Chen, 2012; van der Burg, Awh, & Olivers, 2013; Watanabe & Shimojo, 2001). The underlying mechanisms in our results (rightward-biased attention enhancing multisensory integration in the right hemispace) and those reported by Takeshima and Gyoba’s results (an advantage in the processing linguistic stimuli presented in the right over the left hemispace) are therefore different.

### Similar sound-induced flash illusion in the two hemispaces

To date, surprisingly few studies have deliberately examined whether multisensory integration is symmetrical in the two hemispaces when attention is manipulated. To the best of our knowledge, the only example comes from the phenomenon of the sound-induced flash illusion: A visual flash may be perceived as two flashes when it is accompanied by two auditory beeps, which is a result of binding visual and auditory information together (Shams et al., 2000, 2002; Wozny et al., 2008). In a study by Kamke, Vieth, Cottrell, and Mattingley (2012), the visual flash was presented on either the left or the right (8° from central fixation), while the beeps were presented from loudspeakers situated on both sides of the monitor. Kamke et al. then used transcranial magnetic stimulation (TMS) to deactivate one of the brain areas associated with attention—specifically, the right angular gyrus. The results demonstrated that the probability of perceiving the illusion was reduced by around 20 % in the trials after TMS. Kamke et al. suggested that the integration of the flash and beeps in the perception of the flash illusion was boosted by attention. Nevertheless, the results also demonstrated that the probabilities of perceiving the illusion were similar when the flash was presented in either right or left hemispace, both before and after TMS (see also Innes-Brown & Crewther, 2009).

It should be noted that in Kamke et al.’s (2012) study, the sound-induced flash illusion still occurred on 40 %–45 % of the trials after TMS. That is, even though the illusion can be reduced by deploying TMS over the right angular gyrus, which is associated with attention, it is still a robust effect irrespective of this manipulation of attention. In addition, the right angular gyrus involves multiple functions other than attention (see Seghier, 2013), and some of them likely influence the participants’ performance in the sound-induced flash illusion task. These relevant functions include number processing (to count the number of flashes), memory retrieval (to report the number of flashes after the stimulus presentation), and conflict resolution (to selectively respond to visual flashes rather than auditory beeps). It therefore seems premature to conclude that the modulatory role of right angular gyrus on the sound-induced illusion was simply attributable to attention. Furthermore, a behavioral study of the sound-induced flash illusion has demonstrated that when the participants were instructed to either focus or divide their attention between the visual and/or auditory modalities, the parameters estimated by Bayesian models associated with audiovisual binding were similar in the two conditions (Odegaard et al., 2016).

### A possible mechanism of the modulation of rightward-biased attention on multisensory processing

**Fig. 4:**
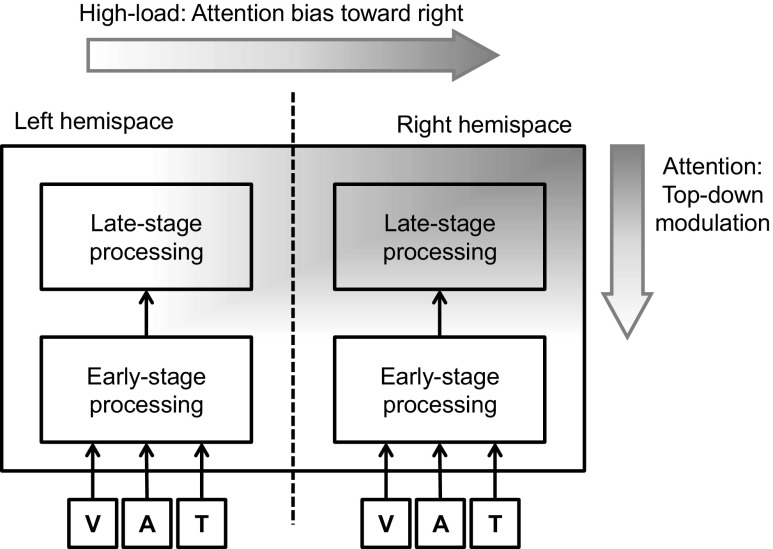
Schematic figure capturing how spatial attention modulates multisensory integration. Darker gray shading represents a greater probability of attentional distribution/modulation. In the pathways of human information processing, multisensory signals interacting at a later rather than an earlier stage would be more susceptible to the top-down modulation of attention. In addition, attention is likely to be biased toward the right hemispace when the task loading is high. Taken together, the hypothesis of asymmetrical attentional modulation is that multisensory processing that requires attention would be more pronounced when the stimuli are presented in right rather than left hemispace (the upper-right corner). V, visual sensory input; A, auditory sensory input; T, tactile sensory input

Taken together, the asymmetrical effects of audiovisual integration are currently observed only in the crossmodal facilitation of masked visual target identification (Chen & Spence, 2011), but not in the sound-induced flash illusion (Innes-Brown & Crewther, 2009; Kamke et al., 2012). By comparing the two experimental paradigms, we propose that at least two factors may be critical regarding whether any given example of multisensory processing would be susceptible to the rightward bias of attention (see Fig. 4). The first one is the level of processing at which the visual and auditory information interact, and the second is the perceptual loading of the participant’s task.

It has been suggested that the sound-induced flash illusion occurs at an early perceptual stage. This is because this illusion is associated with brain activity 35–65 ms after the onset of the flash (Shams, Iwaki, Chawla, & Bhattacharya, 2005), and also with brain activity in the primary visual cortex (Watkins, Shams, Josephs, & Rees, 2007; Watkins, Shams, Tanaka, Haynes, & Rees, 2006). A recent patient study demonstrated that the occurrence of the sound-induced flash illusion is associated with damage in the left and right hemispheres that leads to visual-field deficits, but not with damage in the right hemisphere that leads to left neglect syndrome (Bolognini et al., 2016). Hence, even if the flashes and beeps exogenously capture attention toward the location where they are presented, attention likely does not modulate their integration in a top-down fashion, given that multisensory processing might have been completed. On the other hand, the crossmodal facilitation in the backward-masking task is thought to occur at the level of the object representation. The latter effect has been shown to be susceptible to at least one cognitive factor—that is, the probability of the target letter and the simultaneous tone co-occurring (see Exp. 4 of Chen & Spence, 2011). Therefore, it is possible that the kinds of multisensory processing that occur at a later, rather than an early, processing stage would be more susceptible to the modulation of top-down attention (see Macaluso et al., 2016).

The second possibility pertains to the perceptual load of the information processing associated with the task. In the sound-induced flash illusion, the visual stimulus is a simple flash; by contrast, the visual stimuli in Chen and Spence’s (2011) study consisted of two successive letters presented within a short temporal interval. The perceptual load should therefore be higher for letters than for flashes. Given that attention is more likely to show up as a preferential biasing toward the right hemispace when the perceptual load is high (Eramudugolla et al., 2011; Peers et al., 2006; Pérez et al., 2009), the asymmetrical effect of multisensory integration attributed to the rightward biasing of attention should therefore be more likely to occur in a higher- than in a lower-load task.

In summary, of the two multisensory effects that have been reviewed here, one has been shown to be asymmetrical (crossmodal facilitation in the backward-masking paradigm), whereas the other has not (the sound-induced flash illusion). The crossmodal facilitation effect in the backward-masking paradigm, suggested to occur at the level of object representation, was either prioritized or only significant in the right rather than the left hemispace. On the other hand, the sound-induced flash illusion, which is plausibly an effect of early-level multisensory integration, was symmetrical in the two hemispaces. In addition, a task that is more demanding seems prone to bias the participants’ spatial attention toward the right hemispace. The rightward biasing of spatial attention would seem to offer a promising testable signature with which to dissociate examples of multisensory processing that are susceptible to the top-down modulation of attention from those that are not.

## Conclusions

Previous studies of attention have suggested that attentional mechanisms in the right hemispace are superior to those in the left in the visual, auditory, and tactile modalities. The results of Spence et al.’s (2001) multisensory prior-entry study demonstrate that this rightward bias of spatial attention can be extended to the case of multisensory processing. Furthermore, by reanalyzing the crossmodal facilitation of visual masking by sound reported by Chen and Spence (2011), we suggest that the rightward bias in attention leads to an asymmetrical effect of multisensory processing that requires attention; specifically, it is more pronounced in right than in left hemispace. By contrast, the sound-induced flash illusion, representing a case of early multisensory integration that may occur preattentively, was symmetrical across both hemispaces.

The advantage of utilizing the nature of rightward-biased attention in studies of multisensory processing lies in the fact that the attentional modulation is revealed by the same multisensory stimuli presented in left versus right hemispace, so it is not necessary to manipulate the task difficulty or perceptual load (e.g., Eramudugolla et al., 2011). This approach may, then, be especially suitable for those experimental settings in which the multisensory stimuli are presented only very briefly and need to be processed very rapidly so as to avoid crosstalk between the two hemispheres. Finally, the possible alternative explanation of the right-side advantage in terms of lateralized cognitive function in the left hemisphere (such as for linguistic and temporal processing) should be cautiously ruled out. Our suggestion is that rightward-biased attention provides a novel testable signature with which to probe the role of top-down attention in multisensory processing in future studies.

## Footnotes

1 Using the term *multisensory processing* in the present article follows the definition put forward by Stein et al. (2010, p. 1719), that “... processing involve more than one sensory modality but not necessarily specifying the exact nature of the interaction between them.” Given the fact that attention may involve various types of multisensory processing that would not always lead to an outcome of multisensory integration (i.e., forming a new multisensory representation), we therefore agree with using *multisensory processing* to cover these heterogeneous phenomena.

2 Later studies, however, suggested that, instead of a specific mechanism lateralized to the left hemisphere, linguistic processing may simply be embedded in a general neural network for auditory processing. In this case, the processing of verbal stimuli is distributed in both hemispheres, depending on the area associated with a particular auditory cue (e.g., Friederici & Alter, 2004; Güntürkün, Güntürkün, & Hahn, 2015; Zatorre & Gandour, 2008).

3 The *ventriloquism aftereffect* refers to the phenomenon that, after adapted to a spatially discrepant pair of visual and auditory stimuli, the spatial representation of the latter should be realigned toward the location of the visual stimulus if they were integrated during adaptation. Such audiovisual spatial realignment would remain after adaptation, and therefore is called the *ventriloquism aftereffect*.

4 Extinction between vision and touch provides a critical condition regarding how the spatial frames of reference in the two sensory modalities are mapped in patients. In di Pellegrino et al.’s (1997) study, crossmodal extinction was demonstrated when the tactile stimulus was presented on the left hand, whereas the visual stimulus (a movement of the experimenter’s finger that looked as if it was going to touch the patients’ finger) in front of the right hand. Two additional conditions were also tested for comparison: In one condition, the tactile stimulus remained on the left hand and the visual stimulus was at the same spatial location as in the previous condition; however, now the patient’s right hand was placed behind his back. In this condition, no crossmodal extinction was observed (i.e., the patient detected the tactile stimulus on the left hand correctly; see also Làdavas, di Pellegrino, Farnè, & Zeloni, 1998). In the other condition, the patient was asked to cross his hands, so now the tactile stimulus was on the left hand (in right hemispace) and the visual stimulus was near the right hand (in left hemispace). In this condition, crossmodal extinction of the tactile stimulus on left hand remained. Taken together, these results suggest that the patient’s peripersonal spatial frame was hand-centered, and therefore crossmodal extinction was not induced by a visual stimulus presented far from the right hand, and not influenced by the hands’ locations in terms of the external space frame (di Pellegrino et al., 1997; Làdavas, Berti, Ruozzi, & Barboni, 1997; Làdavas et al., 1998). However, a later study demonstrated that crossmodal extinction was modulated by the manipulation of an external spatial frame: In Kennett, Rorden, Husain, and Driver’s (2010) study, a tactile stimulus on the left hand was extinguished when an LED light was presented near the right hand; however, crossmodal extinction was eliminated when the patient turned her head toward the left, leading to the tactile stimulus on the left hand now spatially to overlap the LED in the right hemispace. This result therefore suggests that the patient utilized an external spatial frame to map the visual and tactile signals on the hand, which is plausibly mediated by proprioceptive perception (see also Kennett, Eimer, Spence, & Driver, 2001; Kennett, Spence, & Driver, 2002; Moseley, Gallace, & Spence, 2009).

5 In Chen and Spence’s (2011) study, the visual stimuli were 18 uppercase letters, excluding the letters F, G, I, L, O, P, Q, and V. The mask for a given target could be any one of the letters used except the target itself or the next letter in the alphabet. The letters were presented in Arial font, which ranged in size from 0.79° × 1.28° to 1.19° × 1.28° (Width × Height). Both the target and mask letters were presented for a duration of 40 ms. The auditory stimulus consisted of a 1000-Hz pure tone, presented simultaneously with the onset of the target and lasting 27 ms. This free-field sound was presented at 54 dB SPL (the background SPL in the experimental chamber was 48 dB). Four loudspeakers were used, two of which (one above and the other below the monitor) were aligned with the left target location, and the other two were aligned with the right target location (at 7.17° left or right from the midline). In each trial, a fixation cross (0.69° × 0.69°) was presented for 1,012 ms and then immediately followed by the target letter; various ISIs (0, 13, 27, 40, 80, and 133 ms) were inserted between the offset of the target letter and the onset of a mask letter. In this case, the 0-ms ISI refers to the condition in which the target letter was immediately followed by the mask letter. The participants were instructed to report the identity of the first letter they saw in each trial (with a chance performance of 6 %), so that the presentation of the tone did not provide any task-relevant information regarding the letter identification response that they would have to make.

6 Nevertheless, such an exogenous orienting of spatial attention should not be elicited by the auditory cue alone, for the following reasons: In Experiment 1, the auditory cue was presented from all four speakers, placed at the four corners. That is, the sound should have been perceived at the center if it was not ventriloquized by the visual target toward left or right. In Experiment 5, the auditory cue was presented from either the two speakers aligned with the left or right location of the visual target. If spatial attention had been oriented by the auditory cue, the effect elicited by the advantage of rightward-biased attention should have been observed in the congruent condition when the visual target was presented in the right hemispace, and an inhibitory effect should have been seen in the incongruent condition when the visual target was presented in the left hemispace, as compared to the no-sound condition. However, this was not the case: The spatial congruency effect was only observed when the visual target was presented in the right hemispace. When these findings are taken together, attentional orienting elicited by the auditory cue alone cannot explain the results observed in Chen and Spence (2011).
